# Individual and family preferences of job qualities matter: association between face needs, locked-in job status, and burnout among high-tech workers in Taiwan

**DOI:** 10.1186/s12889-021-11269-8

**Published:** 2021-06-28

**Authors:** Feng-Jen Tsai, Ruey-Yu Chen, Hsin-Jou Chen

**Affiliations:** 1 Master Program in Global Health and Development, College of Public Health, Taipei Medical University, Taipei, Taiwan; 2 PhD Program in Global Health and Health Security, College of Public Health, Taipei Medical University, Taipei, Taiwan; 3 School of Public Health, College of Public Health, Taipei Medical University, Wu-Hsing Street, 250, Taipei City, 110 Taiwan

**Keywords:** Locked-in job, Burnout, Socially-oriented job preference, Face, Taiwan

## Abstract

**Background:**

Studies on the health impacts of being locked in a job are primarily conducted in Western countries, with the theory based on the value of individualism. But the socially-oriented concerns should be considered in workers’ locked-in status in Chinese society. So the current study aims at evaluating socially-oriented concerns on workers’ locked-in status in Taiwan.

**Methods:**

Anonymous surveys were conducted with 1102 workers at high-tech companies in Taiwan from October 2015 to January 2016 to assess their “face” needs-- a sociological concept linked to the dignity, prestige, and reputation that a person has in terms of their social relationships, locked-in status of the job, and burnout. In addition to being separated into three groups by lock-in score, participants were categorized by the conflict of preference of the job between themselves and their family. Chi-square, ANOVA, Pearson correlation, and regression tests were conducted.

**Results:**

Among the 1102 participants, 18% had jobs that they did not prefer but their family preferred. Participants with higher face needs and higher locked-in status had a significantly higher risk of developing personal and work-related burnout. However, the analysis using “locked-in job conflict of preference between themselves and their family” showed a more coherent result. Participants with a job which “self does not prefer but family do” had twice the risk of having personal and work-related burnout (OR = 2.03 and 2.34, respectively). Participants with a job which neither themselves nor their family prefer had four times the risk of having personal and work-related burnout (OR = 4.10 and 4.17, respectively).

**Conclusions:**

The current study suggests an importance in considering a socially-oriented job preference in locked-in status evaluations within the Chinese culture. Workers’ whose locked-in status preference conflicted with their family’s preference showed a significantly negative impact on their health.

**Supplementary Information:**

The online version contains supplementary material available at 10.1186/s12889-021-11269-8.

## What this paper adds

### What is already known about this subject?

Locked-in status refers to a workers’ non-preference toward his or her job that is harmful to workers’ well-being. However, current studies were conducted only in Western countries with the theory based on the value of individualism.

### What are the new findings?

Contrary to the individually-oriented approach of interacting with the living environment (or life sphere) in Western countries, individuals in Eastern countries generally take a socially-oriented approach to interact with their life sphere. Furthermore, the concern of “face” is one of the more important concepts in an individuals’ decision on their relationship with others in Chinese culture. The current study argues the need to consider a socially-oriented job preference in locked-in studies. Furthermore, study results demonstrated that participants’ face needs and locked-in status were significantly associated with personal and work-related burnout. Participants with higher face needs had a significantly higher risk of developing personal and work-related burnout. Similarly, participants with a higher locked-in status had a significantly higher risk of developing personal and work-related burnout.

### How might this impact on policy or clinical practice in the foreseeable future?

The current study recommends a culture-sensitive policy in workplace mental health program. Furthermore, there is an additional need to understand the impact of socially-oriented job preferences on job performance and workers’ long-term health.

## Background

Historically, being “locked-in” among employees has referred to a situation in which workers have difficulty transiting to another equitable job in labor studies [1–3]. The primary concept of such a situation is the non-preference toward one’s job, so the worker does not want to remain in the current workplace [3]. To remain in the non-preferred job, despite wanting to change companies or workplace, likely causes a strain on workers and leads to impaired well-being [2–5]. Though research on the health impacts of locked-in jobs is comparatively rare, the existing studies demonstrate that people in non-preferred jobs tend to report poor psychological well-being, including burnout [2]. Recently, Stengard added the element as “perceived employability” to being locked-in with the argument that a revolving situation of a non-preferred occupation frequently requires a complete career re-orientation, which is difficult. Therefore, perceived employability plays a role in the locked-in status of employees. Additionally, their studies, based on longitudinal designs, also showed the negative impact of locked-in status on well-being among workers [6, 7].

However, previous studies were conducted only in Western countries with the theory based on the value of individualism. Studies have suggested that even though the benefit of a voluntary job transition such as increased salary or self-esteem was clear, employees would occasionally decide to stay in their current job despite the availability of other suitable jobs for several reasons [8, 9]. With the exception of the satisfaction of their current job, the fit between work and private life and social relationships with co-workers were listed as the primary reasons for staying [10]. These findings suggest the broader work-related context, including that factors that are more indirectly connected to the job rather than the work tasks themselves, could be an important influence for remaining in a job.

Contrary to the individually-oriented approach of interacting with the living environment (or life sphere) in Western countries, individuals in Eastern countries generally take the socially-oriented approach to interact with their life sphere [11–13]. The founder of Indigenized Chinese psychology, Kuo-shu Yang, proposed a four-part theory of the Chinese self to explain how Chinese individuals build their own identity and interact with others [14, 15]. He argued that the Chinese self is composed of both the individually-oriented and socially-oriented self, with the latter further dividing into the relationship-oriented, family (group)-oriented, and other-oriented self. According to the definition, “the individually-oriented self is a combination of a tendency toward high personal autonomy and low homonymy that emphasizes an individual’s personal achievement, performance, uniqueness, and autonomy” [16], while “the socially-oriented self is a combination of a tendency toward high homonymy (with the surrounding environment) and low personal autonomy, which emphasizes that this type of self tends to attach importance in order to maintain harmonious interpersonal relationships, accountability, and responsibility, and requires appropriate personal behaviors such that individuals position themselves according to their relationship with others” [16]. As an individual raised in a collectivist culture, concerns of family and others are fundamental in a variety of individual decisions, including decisions related to work.

The concern of face is one of the most important concepts in individuals’ decisions concerning the relationship with others in Chinese culture [11, 17]. Contrary to the concept of face in the Western culture as politeness, in Chinese culture it refers to a sociological concept that is linked to the dignity, prestige, and reputation that a person has in terms of their social relationships [18, 19]. While job title and industry are related to the social recognition of workers in Eastern culture, the decision of taking a job or transferring to another job is highly connected with the concept of face of individuals [17]. In addition, Taiwan is a typical Chinese society and, as such, is strongly influenced by the traditional value of filial piety [20, 21]. Many Chinese parents consider the job and career of their adult child to be an issue of face, which represents their achievement in raising their child [22]. They will, therefore, urge their adult child to take a job with a strong social reputation. With these strong cultural influences, we suggest that the socially-oriented concerns should be considered in locked-in evaluations, such that the individual’s family’s job preference also may play a role in workers’ locked-in status in Chinese society. Therefore, we hypothesize that workers in Taiwan might take a job they do not prefer based on the concern of face of both themselves and their parents. Furthermore, the conflict of personal preference and family preference toward one’s job represents the locked-in status of the individual and may lead them to develop burnout syndrome.

In detail, the current hypotheses are:

- Hypothesis 1: The socially-oriented job preference plays an important role in workers’ locked-in status. Additionally, the locked-in status with which the worker’s preference conflicts with their family’s preference will have a negative impact on workers’ health. In other words, the higher the score of lock-in status due to conflicting preference, the higher the risk of burnout among workers.
- Hypothesis 2: Workers’ face needs are positively related to their locked-in status, represented by workers’ preferences and the preference of their family. In other words, workers with higher score of face needs have a higher risk of being locked-in job.

In Taiwan, work in high-technology industries are considered a fashionable job with a potentially high salary and good career future [23]. Additionally, working for a big company is considered to be a good job due to the presumed job security and popular recognition among society, though the working conditions, including salary and work hours, might sometimes be worse than in smaller companies [24]. Therefore, we conducted the current study on high-tech company workers.

## Methods

The current study was conducted using an anonymous self-report questionnaire given to workers at three high-tech companies in Taiwan from October 2015 to January 2016 to assess their face needs, locked-in status of their job, and burnout. Those companies were all in electronic industry which producing computer-related products. And two companies had more than 500 workers while another one had around 300 workers.

The questionnaire was delivered to 2341 workers and 1116 returned their questionnaire surveys (response rate of 47.68%). After excluding 14 questionnaires with more than 10 invalid response items, 1102 questionnaires were included in the final analysis.

The research protocol was approved by the Institutional Review Board of Taipei Medical University (No. N201805098). While it is an anonymous survey, only participants who were willing to participate in the study would return the questionnaire. While the research involved survey without identifiers, answering the survey served as implicit consent. So informed consent was taken from all participants. All methods were carried out in accordance with Declaration of Helsinki.

### Measures

#### Independent variables

Work-related individual characteristics, including job content (engineer, administrator and others), age (from 22 to 64 years old), gender (male/female), education level (high and professional school/college/master and above), marital status (single/married or with partner/others), work experience (years), work level (administrative level or not), work hours per week, and company size (above 500 persons/ less than 500 persons) were all collected for the analyses.

#### Locked-in status

The workplace preference scale from Stengard’s locked-in questionnaire was used in and modified for the study [6, 7]. Workplace preference was measured by questions such as “Is your current occupation the occupation you expected?”; “Is your current occupation the occupation you wish to have in the future?”; “Is the company you work for today the company you expected?”; and “Is the company you work for today the company you want to work for in the future?”

The socially-oriented questions of locked-in status was added as “Is your current occupation the occupation your family (parents, spouse, and relatives) expected?” and “Is the company you work for today the company your family expected?” The responses were evaluated on a 4-point Likert-scale, ranging from 1 = very much disagree to 4 = very much agree.

Information of perceived employability from Stengard’s locked-in questionnaire was also collected for analysis. The perceived employability was measured by a question as “I don’t need to move or rent another additional house for finding another similar work. I can easily find another similar work like the current one.”

#### Face needs

For evaluating the construct of face needs, we used the “face needs questionnaire” developed and validated by a Chinese psychologist [25–27]. Cronbach’s α was 0.84 for our study. The questionnaire of face covered two dimensions: (1) concern about face of one’s self (14 items) and (2) concern about face of others (seven items). The detail information of the items was provided in the supplementary file. The responses were evaluated using a 5-point Likert-scale ranging from 0 = very much disagree to 5 = very much agree. The higher scores represent a higher face need of participants.

#### Burnout

Burnout was used as a health outcome indicator in the current study. The Chinese version of the 21-item Copenhagen Burnout Inventory (C-CBI) was used to measure the three domains of burnout: personal burnout, work-related burnout, and client-related burnout [28]. In the current study, only the personal (five items) and work-related burnout (five items) items were measured among workers. Per the CBI, personal burnout is defined as “the degree of physical and psychological fatigue and exhaustion experienced by the person”, while work-related burnout is “the degree of physical and psychological fatigue and exhaustion perceived by the person as related to his/her work” [29]. The responses were evaluated using a 5-point Likert-scale from 0 = never to 5 = always. The C-CBI was validated by Taiwaness researchers [30]. And the Cronbach’s α was 0.93 for our study.

We used expert review approach to obtain the content validity of all the variables and measure except standardized questionnaire as Face-needs and burnout. Six related experts were invited for the review. And the Item-level Content Validity Index (I-CVI) of each item was above 0.78 and used in the study.

### Data analyses

Two approaches were used to calculate the locked-in status of participants. The scores of participants’ preference and their family’s preference of the job were summed to comprise the locked-in scores. Then, the locked-in scores were divided into three groups for analysis. Participants were also categorized by the conflict of the preference of the job between themselves and their family as “locked-in job conflict of preference between themselves and their family” status. Participants were divided into four groups as “both prefer”, “self prefers but family does not”, “self does not prefer but family does”, and “both do not prefer”.

Three different approaches were used to perform the analyses. For summary statistics, a chi-square test was used to compare individual characteristics including age, gender, educational status, marital status, job title, work level, work year, working hours per day, and company size by “locked-in job conflict of preference between themselves and their family” status. An ANOVA was used to compare the means of face needs, including face of self and face of others, burnout, including personal and work-related burnout, and self-evaluated employability between “locked-in job conflict of preference between themselves and their family” status. A Pearson correlation analysis was used to evaluate the association between face needs and locked-in status.

Personal burnout and work-related burnout were defined as dichotomous variables with the cut-point as the mean. A logistic regression was then adopted to estimate the relationship between face needs, locked-in status, and burnout after adjusting for confounding variables. Variables that were significantly associated with personal and work-related burnout in the bivariate analysis including age, gender, educational status, marriage status, job title, work year, number of hours worked per day, company size, and self-perceived employability were adjusted for in the regression model (supplementary). The regression model was also run to evaluate the associations between face needs, “locked-in job conflict of preference between themselves and their family” status, and burnout.

The odds ratios (OR) and related 95% confidence intervals (CIs) were also calculated. The significance level was set at 0.05. All analyses were performed using SPSS, Version 18.0.

## Results

### Comparison of individual characteristics between “locked-in job conflict of preference between themselves and their family” status groups

The comparison of individual characteristics between “locked-in job conflict of preference between themselves and their family” status groups using a chi-square test are shown in Table 1. In general, majority of the participants were single (57.17%), male (59.44%), aged 30 to 39 years old (53.36%), and had obtained an educational degree above college (97.83%). Moreover, 68.78% of the participants were engineers without an administrative position. While 48.55% of participants had only worked for less than 5 years, 54.72% of them worked over 40 h per week, and 83.58% of them worked for a large company with more than 500 employees.

**Table 1:**
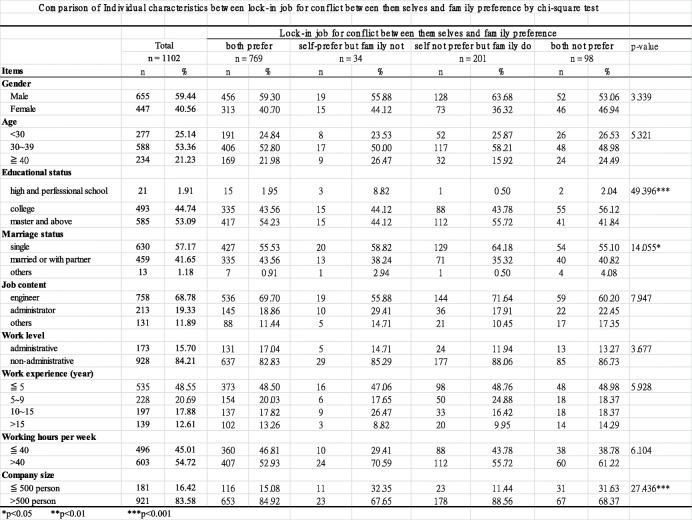
Comparison of Individual characteristics between lock-in job for conflict between themselves and family preferences by chi-square test

Among the 1102 participants, 18% had a job that they did not prefer but their family preferred and 9% of them had a job which neither themselves nor their family preferred. Educational status, marriage status, and company size were significantly different between “lock-in job conflict of preference between themselves and their family” status groups. Those with an educational level at Master’s degree and above comprised the largest group in the “self does not prefer but family does” group (55.72%), followed by the “both prefer” group (54.23%), the “self prefers but family does not” group (44.12%), and the “both do not prefer” group (41.84%). Participants who were married were most represented in the “both prefer” group (43.56%), followed by the in “both do not prefer” group (40.82%), the “self prefers but family does not” group (38.24%), and finally in the “self does not prefer but family does” group (35.32%). The percentage of participants who worked in big company was the highest in the “self does not prefer but family does” group (88.56%), followed by the “both prefer” group (84.92%), the “both do not prefer” group (68.37%), and the “self prefers but family does not” group (67.65%). The other factors were not significantly different between the groups.

### Comparison of face needs, burnout, and self-perceived employability between “locked-in job conflict of preference between themselves and their family” status groups

Comparisons of face needs, burnout, and employability between “locked-in job conflict of preference between themselves and their family” status groups using an ANOVA are shown in Table 2. Face needs, including concern of face of one’s self and concern of face of others, burnout, including personal burnout and work-related burnout, and self-perceived employability were all significantly different between groups. The mean of face needs was highest in the “both prefer” group (70.7), then in the “self does not prefer but family does” group (70), then in “both do not prefer” group (67.59), and finally in the “self prefers but family does not” group (66.68). The concern of face of one’s self was highest in the “both prefer” group, then in “self does not prefer but family does” group, then in the “both do not prefer” group, and then in “self prefers but family does not” group. Contrarily, the mean of concern of face of others was the highest in the “self does not prefer but family does” group, then in the “both prefer” group, then in the “both do not prefer” group, then in the “self prefers but family does not” group.

**Table 2:**
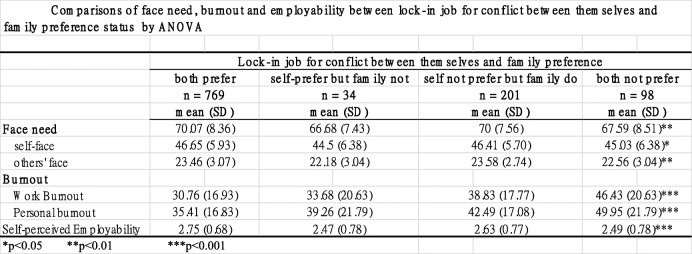
Comparisons of face need, burnout and employability between lock-in job for conflict between themselves and family preferences status by ANOVA

Personal burnout was the highest in the “both do not prefer” group, then in the “self does not prefer but family does” group, then in the “self prefers but family does not” group, then in the “both prefer” group. Similarly, work-related burnout was the highest in the “both do not prefer” group, then in the “self does not prefer but family does” group, then in the “self prefers but family does not” group, then in the “both prefer” group.

Self-perceived employability was highest in the “both prefer” group, then in the “self does not prefer but family does” group, then in the “both do not prefer” group, then in the “self prefers but family does not” group.

The Pearson correlation analysis showed that participants’ face needs are not correlated with their locked-in status as evaluated by both approaches.

### Associations between face needs, locked-in job status, and burnout

The associations between face needs, locked-in job status, and burnout when using a regression are shown in Table 3. Workers’ marital status, job title, hours worked per day, and their company size, self-perceived employability, face needs, and locked-in job status were all significantly associated with personal and work-related burnout.

**Table 3:**
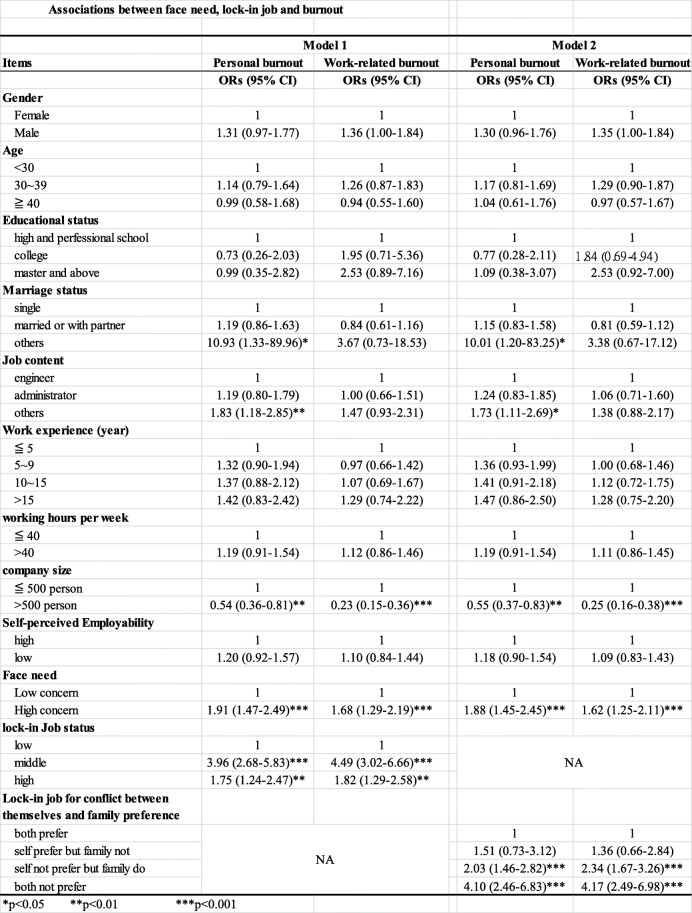
Association between face need, lock-in job and burnout

For model 1, the tertile locked-in scores were used for the analysis and marital status, job title, company size, face needs, and locked-in status were all significantly related to personal burnout. Participants with a marriage status marked as other (divorce or widowed) and job title marked as other had a significantly higher risk of developing personal burnout than single participants or those whose job title was “other” (i.e. not an engineer or in administration) (OR = 10.93 and OR = 1.83, respectively). Participants who worked in a big company had a significantly lower risk of having personal burnout compared to participants who worked in smaller sized companies (OR = 0.54). Importantly, participants with high face needs had a significantly higher risk of developing personal burnout (OR = 1.91). Moreover, participants with a high locked-in job status had a significantly higher risk of having personal burnout (OR = 1.75), while participants with a middle locked-in job status had a four times greater risk of having personal burnout (OR = 3.96).

Participants who worked for a big company had a significantly less chance of developing work-related burnout (OR = 0.23), while those with high face needs had a significantly higher risk of developing work-related burnout (OR = 1.68). Moreover, participants with a high locked-in job status had a significantly higher risk of having personal burnout (OR = 1.82) and participants with a middle locked-in job status had a four times greater risk of having personal burnout (OR = 4.49).

For model 2, which used “locked-in job conflict of preference between themselves and their family” status for the analysis, marriage status, job content, company size, face needs, and “locked-in job conflict of preference between themselves and their family” status were all significantly related to personal burnout. Participants with a marriage status listed as other (i.e. divorced or widowed) and job content described as other had a significantly higher risk of developing personal burnout than single participants (OR = 10.01 and OR = 1.73, respectively). Participants who worked in a big company had a significantly lower risk of having personal burnout than participants who worked in smaller sized companies (OR = 0.55) and participants with high face needs had a significantly higher risk of developing personal burnout (OR = 1.88). Moreover, participants with a job status of “self does not prefer but family does” had twice the risk of having personal burnout (OR = 2.03), while participants with a job that both themselves and their family do not prefer had a four times greater risk of having personal burnout than those with a job that both themselves and their family prefer (OR = 4.10).

Participants who worked in a big company had a significantly less chance of work-related burnout (OR = 0.25), while participants with high face needs had a significantly higher risk of developing work-related burnout (OR = 1.62). Moreover, participants with “locked-in job conflict of preference between themselves and their family” status of “self does not prefer but family does” had twice the risk of having work-related burnout (OR = 2.34) and participants with a job that neither themselves nor their family preferred had a four times greater risk of having work-related burnout (OR = 4.17).

## Discussion

To the best of our knowledge, the current study is the first to aim at evaluating the impact of special concerns in the Chinese culture, namely the concept of face on locked-in status and burnout among workers. Also, this is the first study to suggest the need to consider the socially-oriented job preference in locked-in studies. The current results demonstrated that participants’ face needs and locked-in status were significantly related to their personal and work-related burnout. Participants with higher face needs and, independently, higher locked-in status had a significantly higher risk of developing personal and work-related burnout. The study result confirmed our hypothesis 1 and hypothesis 2.

As confirmed by the results of the current study, it is not surprising that participants with a higher locked-in status showed a significantly higher risk of developing personal and work-related burnout. And the study finding was similar with Stengard’s and Fahlen’s studies [5, 6]. Interestingly, however, participants with a middle locked-in status had the highest risk of developing personal and work-related burnout. Their risk is even higher than workers with a high locked-in status. This phenomenon may be due to the fact that two thirds of our participants whose preference was in conflict with their family were categorized in the middle locked-in group. The analysis using “locked-in job conflict of preference between themselves and their family” status showed a more coherent result for the risk of developing burnout syndrome among workers. Participants with a job status as “self does not prefer but family does” had twice the risk of having work-related burnout, while participants with a job that neither they nor their family preferred had a four times greater risk of having work-related burnout. These result suggest that workers’ preference toward their job played the primary role in protecting their health. Even if the worker has a high socioeconomic status job that everyone admires, if s/he does not like the job, their feeling of being trapped at work will lead them to develop burnout syndrome. Additionally, these results support the notion that socially-oriented concerns should be considered in locked-in evaluations in Chinese society. These finding may also generalize to other Asian countries with a patriarchal culture. And we suggest to conduct relevant comparative studies in multi-ethnic Western countries in the future in order to further understand the impact of conflict between individual and their family regarding their job choice on workers’ health. In the meantime, we suggest the introduction of mental health program regarding the issue in the workplace in order to reduce the negative impact of such issue on workers’ health.

The current results demonstrated that participants with higher face needs had a significantly higher risk of having personal and work-related burnout. Although the correlation between face needs and locked-in status was not demonstrated, workers who stayed in a job which “self does not prefer but family does” were those who tended to have high face needs. There were 18% participants in our study who were locked into the job that they did not prefer but their family preferred. Given these participants had the highest concern of face of others, there is a high possibility that they stayed in a job they did not prefer in order to preserve face of their parents or families. In other words, those workers who had high face needs and a high concern of face of others were locked in job due to their concern of face. As such, workers who had high face needs demonstrated a significantly higher risk of developing personal and work-related burnout.

Interestingly, participants (69.78%) who reported that both themselves and their family prefer their job also reported similarly high face needs compared to the workers stayed in their job in which they did not prefer but their family did. Interestingly, even though the percentage of those who were married and the percentage of those who worked in smaller companies were higher in the “both prefer” group, the socio-economic background of those two groups was similar. The majority of those two groups were comprised of single young men with an educational level of a Master’s degree or above who were working as engineers. In addition, nearly 50% of them had work experience of less than 5 years. It is plausible that participants in “both prefer” group had convinced themselves that they liked the job because they felt proud about their good socio-economic status and enjoyed the feeling of “having face” for themselves and their family. Additional research is needed to understand the reason behind this phenomenon.

Several limitations were noted in the current study. First, the response rate was comparatively low. The non-responders may be the workers with higher levels of work stress and there may be an under-reporting of the locked-in status among high-tech company workers. Secondly, relationships should only be considered as correlational rather than causal due to the cross-sectional design. Finally, it is important to not discount a potential reporting bias due to the self-report study design.

## Conclusion

In conclusion, the current study indicated the importance of considering the socially-oriented job preference in locked-in status evaluations in Chinese society. Both participants’ face need and locked-in status were significantly associated with their personal and work-related burnout. Participants with higher face needs had a significantly higher risk of developing personal and work-related burnout and workers’ locked-in status who had a conflicting preference compared to that of their family demonstrated a significantly negative impact on their health. Additional studies are recommended to understand the impact of the socially-oriented job preference on job performance and workers’ long-term health.

## Supplementary Information

**Additional file 1: Supplementary 1.** Detail information of the “Face Need Questionnaire”. **Supplementary 2.** Correlation table for variables by Pearson analysis.

## Data Availability

The datasets used and/or analyzed for the current study are available from the corresponding author upon reasonable request.

## Abbreviations

High-tech: High technology
CBI: Copenhagen Burnout Inventory
OR: Odds ratio
CIs: Confidence intervals

## References

[1] Greenhaus JH, Callanan GA (2013). Career dynamics. Handbook of psychology: Industrial and organizational psychology.

[2] Aronsson G, Goransson S (1999). Permanent employment but not in a preferred occupation: psychological and medical aspects, research implications. J Occup Health Psychol.

[3] Fouad NA, Bynner J (2008). Work transitions. Am Psychol.

[4] Muhonen T (2010). Feeling double locked-in at work: Implications for health and job satisfaction among municipal employees. Work.

[5] Fahlen G, Goine H, Edlund C, Arrelov B, Knutsson A, Peter R (2009). Effort-reward imbalance, “locked in” at work, and long-term sick leave. Int Arch Occup Environ Health.

[6] Stengård J, Bernhard-Oettel C, Berntson E, Leineweber C, Aronsson G (2016). Stuck in a job: being “locked-in” or at risk of becoming locked-in at the workplace and well-being over time. Work Stress.

[7] Stengård J, Berntson E, Leineweber C, Bernhard-Oettel C. Who gets stuck in their workplaces? The role of matching factors, between individual and job, and demographics in predicting being locked in. Scand J Work Organ Psychol. 2019;4(1). 10.16993/sjwop.56.

[8] Ng TWH, Sorensen KL, Eby LT, Feldman DC (2007). Determinants of job mobility: a theoretical integration and extension. J Occup Organ Psychol.

[9] Ng TWH, Feldman DC (2007). Organizational embeddedness and occupational embeddedness across career stages. J Vocat Behav.

[10] Mitchell TR, Holtom BC, Lee TW, Sablynski CJ, Erez M (2001). Why people stay: using job embeddedness to predict voluntary turnover. Acad Manag J.

[11] Hwang KK (1987). Face and Favor: The Chinese Power Game. Am J Sociol.

[12] Yang K-S (2000). Monocultural and cross-cultural indigenous approaches: the royal road to the development of a balanced global psychology. Asian J Soc Psychol.

[13] Yang K-S (1981). Social orientation and individual modernity among Chinese students in Taiwan. J Soc Psychol.

[14] Yang KS, Lu L (2005). Social- and individual-oriented self-actualizers: conceptual analysis and empirical assessment of their psychological characteristics. Indigen Psychol Res Chin Soc.

[15] Yang KS. Chinese Social Orientation: An Integrative Analysis. In: Lin TY, Tseng WS, Yeh YK (Eds). Chinese Societies and Mental Health. Hong Kong: Oxford University Press; 1995. p. 19–39.

[16] Yang KS (2004). A theoretical and empirical analysis of the Chinese self from the perspective of social and individual orientation. Indigen Psychol Res Chin Soc.

[17] Chu R-L (1989). Face and achievement: the examination of social oriented motives in Chinese society. Chin J Psychol.

[18] Han K-H (2016). The feeling of “face” in Confucian society: from a perspective of psychosocial equilibrium. Front Psychol.

[19] Kim JY, Nam SH (1998). The concept and dynamics of face: implications for organizational behavior in Asia. Organ Sci.

[20] Yi C-C, Lin J-P (2009). Types of relations between adult children and elderly parents in Taiwan: mechanisms accounting for various relational types. J Comp Fam Stud.

[21] Coombs LC, Sun T-H (1978). Family composition preferences in a developing culture: the case of Taiwan, 1973. Popul Stud.

[22] Chu CR-L, Poston JD, Yang WS, Farris DN (2014). Family Values and Parent–Child Interaction in Taiwan. The Family and Social Change in Chinese Societies.

[23] Investigate the best tech industries in the minds of office workers [https://www.1111.com.tw/news/surveyns/122264/]. Accessed 16 Apr 2021.

[24] Big company has face? Small companies make money? How to choose a programmer’s employment [https://kknews.cc/zh-tw/career/ygoe9qn.html]. Accessed 16 Apr 2021.

[25] Ruey-Ling C (1987). Chinese social interaction: on the operation of face. Chin J Sociol.

[26] Ruey-Ling C (1993). Face and achievement: a probe into social orientation motivation. Chin J Psychol.

[27] Chu R-L (1991). Face, pressure and coping behavior. Nat Sci Council Res Transact.

[28] Yeh W-Y, Cheng Y, Chen C-J, Hu P-Y, Kristensen T (2007). Psychometric properties of the Chinese version of Copenhagen burnout inventory among employees in two companies in Taiwan. Int J Behav Med.

[29] Kristensen TS, Borritz M, Villadsen E, Christensen KB (2005). The Copenhagen burnout inventory: a new tool for the assessment of burnout. Work Stress.

[30] Cheng Y, Guo YL, Yeh WY (2001). A national survey of psychosocial job stressors and their implications for health among working people in Taiwan. Int Arch Occup Environ Health.

