# Supplementary material for: Individual and family preferences of job qualities matter: association between face needs, locked-in job status, and burnout among high-tech workers in Taiwan
Source: BMC Public Health. 2021 Jun 28;21:1241. doi: 10.1186/s12889-021-11269-8 (PMC8240265; doi:10.1186/s12889-021-11269-8)
Supplement: Supplementary file 1 — Additional file 1: Supplementary 1. Detail information of the “Face Need Questionnaire”. Supplementary 2. Correlation table for variables by Pearson analysis. [file 12889_2021_11269_MOESM1_ESM.pdf]

Supplementary 1—Detail information of the "Face Need Questionnaire"

1. Concern about face of one's self
    1.1. When someone criticizes me, it affects me more if done in public than in private.
    1.2. During a discussion, I try not to ask questions because I may appear ignorant in front of others.
    1.3. I keep a low profile because I don't want to make mistakes in front of others.
    1.4. Before I express my opinion in front of others, I will carefully construct it first, to minimize any offense toward others.
    1.5. I downplay my abilities and achievements so that others will not have unrealistic expectations of me.
    1.6. I plan my words and actions carefully to reduce errors.
    1.7. Before commenting on things, I will first say that I may not necessarily be correct.
    1.8. When I interact with others, I care about what they expect of me.
    1.9. I feel hesitant to ask for assistance because I think my request will cause an inconvenience of others.
    1.10. I try not to do things that will cause others to notice me.
    1.11. I don't criticize others because it makes them embarrassed.
    1.12. Before I do things, I carefully observe the actions of others.
    1.13. I do not complain publicly, even if I have been treated unfairly.
    1.14. I try to make myself behave like others in order to meet social standards.

2. concern about face of others
    2.1. Before I do anything publicly, I prepare myself for any possible outcomes.
    2.2. I like to use third parties to resolve disagreements between myself and others.
    2.3. When discussing a problem, I try to let the other person know that I am not accusing him or her.
    2.4. When someone criticizes me, I try to avoid that person.
    2.5. When I make a mistake in front of everyone, I try to keep them from noticing the mistake.
    2.6. "Even if I know someone else is at fault, I am careful not to criticize them"; and "When someone embarrasses me, I try to ignore them".

Supplementary 2—Correlation table for variables by Pearson analysis

		gender	age	educational status	marriage status	job content	work level	work year	work hours per day	company size	Face need	personal burnout	work-related burnout	self-perceived employability	lock-in job status
gender	Pearson correlation														
	p-value														
age	Pearson correlation	.029													
	p-value	.344													
educational status	Pearson correlation	.255	-.198												
	p-value	.000	.000												
marriage status	Pearson correlation	.006	.512	-.176											
	p-value	.838	.000	.000											
job content	Pearson correlation	-.400	.080	-.375	.078										
	p-value	.000	.008	.000	.010										
work level	Pearson correlation	.102	.318	.034	.302	.013									
	p-value	.001	.000	.266	.000	.661									
work year	Pearson correlation	-.007	.649	-.226	.504	.058	.364								
	p-value	.807	.000	.000	.000	.055	.000								
work hours per day	Pearson correlation	.079	.155	-.030	.044	.004	.096	.110							
	p-value	.009	.000	.317	.147	.898	.001	.000							
company size	Pearson correlation	.163	-.202	.369	-.138	-.324	.029	-.041	-.162						
	p-value	.000	.000	.000	.000	.000	.338	.179	.000						
Face need	Pearson correlation	.012	-.134	.018	-.061	-.014	-.113	-.110	-.067	.091					
	p-value	.689	.000	.558	.045	.650	.000	.000	.027	.002					
personal burnout	Pearson correlation	-.096	.064	-.043	.088	.134	.034	.070	.062	-.139	.100				
	p-value	.001	.035	.156	.003	.000	.263	.020	.040	.000	.001				
work-related burnout	Pearson correlation	-.103	.014	-.037	-.006	.118	-.033	-.009	.058	-.222	.082	.642			
	p-value	.001	.652	.224	.848	.000	.268	.756	.055	.000	.006	.000			
self-perceived employability	Pearson correlation	-.006	-.050	.012	-.046	.002	-.046	-.006	.009	.062	-.022	.051	.031		
	p-value	.833	.096	.702	.123	.941	.131	.847	.764	.039	.458	.093	.311		
lock-in job status	Pearson correlation	-.010	.021	-.052	.042	.004	.010	.014	-.060	-.001	.129	-.214	-.249	-.095	
	p-value	.750	.487	.085	.160	.882	.750	.641	.049	.977	.000	.000	.000	.002	
lock-in job for conflict between themselves and family preference	Pearson correlation	.018	-.034	-.006	-.053	.008	-.058	-.024	.037	-.011	-.033	.175	.198	.105	-.558
	p-value	.546	.265	.841	.079	.803	.056	.426	.217	.727	.268	.000	.000	.000	.000
